# Impact of self-directed e-learning on nurses’ competency in arrhythmia interpretation in cardiology

**DOI:** 10.1371/journal.pone.0331043

**Published:** 2025-09-16

**Authors:** Marie Jane de Lima, Naser Jamil, Seema Vazhuthakkadu, Sultan Mosleh, Nezam Al-Nsair

**Affiliations:** 1 Nursing Department, Cardiology complex, Rashid Hospital, Dubai Health, Dubai, United Arab Emirates; 2 Higher Colleges of Technology, Health Science Division, Sharjah, United Arab Emirates; 3 Mutah University, Faculty of Nursing, AL Karak, Jordan; 4 Hind Bint Maktoum College of Nursing and Midwifery (HBMCoNM), Mohammed Bin Rashid University of Medicine and Health Sciences (MBRU), Dubai Health, Dubai, United Arab Emirates; Government Villupuram Medical College and Hospital, INDIA

## Abstract

Accurate electrocardiogram (ECG) interpretation is an essential competency for nurses, particularly in cardiology, where the timely identification of arrhythmias can be lifesaving and significantly impact patient outcomes. Nurses often serve as the first line of clinical observation, making their ability to interpret ECGs critical for early intervention and safe patient care. However, numerous studies have highlighted persistent gaps in ECG interpretation skills among nursing staff, emphasizing the urgent need for effective, accessible educational strategies.

This study aimed to assess the effectiveness of a self-directed e-learning (SDL) package in improving nurses’ knowledge and competency in arrhythmia interpretation within the cardiology department of Rashid Hospital, Dubai. A quasi-experimental, one-group pre-test/post-test design was utilized with a sample of 50 nurses working in Coronary Care Units. Data were collected using a validated, structured questionnaire that included demographic data and ECG interpretation tests. The SDL package covered foundational ECG knowledge, rhythm analysis, and arrhythmia management.

Results showed a statistically significant 15.92% improvement in knowledge following the intervention (t = −6.668, p < .001). A notable correlation was observed with years of experience; nurses with 1–5 years of experience demonstrated the highest improvement (p = .020). No significant differences were found based on gender (p = .234) or area of practice (p = .139).

This study highlights the critical need to strengthen nurses’ arrhythmia interpretation skills and demonstrates that SDL is an effective, flexible, and scalable approach to bridging competency gaps in high-acuity clinical areas such as cardiology.

## Introduction

Electrocardiography (ECG) has become an indispensable diagnostic tool in modern medicine, allowing healthcare professionals to monitor the heart’s electrical activity and detect various cardiovascular conditions. Initially developed over a century ago, the ECG has since been widely adopted as a primary diagnostic method for acute coronary syndrome (ACS) and other cardiac conditions [1,2]. This non-invasive test plays a crucial role in diagnosing life-threatening conditions such as arrhythmias, including ventricular tachycardia and ventricular fibrillation, both of which are leading causes of sudden cardiac death globally [3,4] Given its wide utility, ECG interpretation is an essential skill for healthcare professionals, particularly nurses, who often serve as first responders in critical care settings, such as intensive care units [ICU], emergency departments [ED], and cardiac care units [2–4]

Accurate ECG interpretation is essential for timely diagnosis and intervention in cardiovascular emergencies. The ability to correctly identify abnormal rhythms, such as arrhythmias, can significantly impact clinical decisions and patient outcomes [5]. In particular, rapid interpretation is critical in situations where immediate action is required, such as cardiac arrest or the onset of life-threatening arrhythmias. However, despite the importance of ECG interpretation, studies reveal significant gaps in nurses’ competency in this area, particularly among those working in acute care environments [5,6].

Previous research highlights that nurses’ knowledge and proficiency in ECG interpretation vary widely depending on their clinical experience and training. For example, Tahboub & Yilmaz [3] found that many nurses, particularly those with limited experience, struggled to correctly interpret ECG tracings, which in turn affected their ability to provide timely interventions. Similarly, a recent study identified significant gaps in ECG interpretation skills among healthcare professionals, including nurses, with accuracy rates varying widely across disciplines [7]. These gaps underscore the need for improved educational strategies aimed at enhancing nurses’ ECG interpretation skills.

One promising approach to addressing this competency gap is the implementation of structured educational interventions, such as self-directed learning [SDL] packages. The SDL approaches allow healthcare professionals to learn at their own pace, which can be particularly beneficial in busy clinical environments where time for traditional training may be limited [8]. Moreover, a self-directed interventions significantly enhanced ECG interpretation competency across a diverse range of healthcare professionals [9]. The SDL programs can significantly improve nurses’ ECG interpretation skills, with participants showing marked improvements in both knowledge and practical proficiency after completing the training [10]..

### Significance of the study

This study is significant as it addresses a critical gap in the training and competency of nurses in ECG interpretation, particularly in acute care settings where rapid and accurate diagnosis is essential for patient survival. Cardiovascular diseases, including arrhythmias, remain the leading cause of death worldwide [11], Given the crucial role that nurses play in monitoring and interpreting ECGs in emergency and critical care settings, improving their proficiency in this skill is directly linked to better patient outcomes [3,4]

Despite the clear need for enhanced training, traditional methods such as classroom learning and workshops have limitations, particularly when it comes to fostering long-term retention and application of ECG interpretation skills [5,6] This study explores the use of a self-directed learning package as a flexible and effective alternative to traditional training methods, with the goal of providing evidence for its efficacy in improving nurses’ ECG interpretation competency.

The purpose of this study is to evaluate the effectiveness of a self-directed learning (SDL) package in improving the ECG interpretation skills of nurses working in critical care settings. Specifically, the study aims to assess whether the SDL intervention can enhance nurses’ ability to accurately detect and diagnose arrhythmias, thus improving their overall competency in ECG interpretation. The study also seeks to determine whether demographic factors, such as years of experience, influence the effectiveness of the SDL package.

The study addresses the following research questions:

- What is the baseline level of knowledge and skill in ECG interpretation among nurses working in critical care settings?
- Does the implementation of a self-directed learning package improve nurses’ ability to accurately interpret ECGs and detect arrhythmias?
- How do demographic factors, such as years of clinical experience and area of practice, affect improvements in ECG interpretation skills following the SDL intervention?

## Methods

### Study design

This study employed a quasi-experimental, one-group pre-test/post-test design to evaluate the effectiveness of a self-directed learning (SDL) package on nurses’ knowledge and skill in ECG interpretation, particularly arrhythmia detection.

### Population and sample

The study was conducted between March and May 2023 and included 50 staff nurses working in the Cardiology Units of Rashid Hospital, Dubai, which included the Coronary Care Unit (CCU), High Dependency Unit (HDU), and Cardiac Interventional Unit. Participants were selected through purposive sampling based on their availability during the study period and their clinical involvement in ECG monitoring and interpretation. The inclusion criteria required participants to have at least one year of nursing experience in cardiac units, and they had to provide written informed consent prior to participation.

### Setting

This research was conducted at Rashid Hospital, a 786-bed tertiary care hospital in Dubai, UAE. The study focused on the cardiology department, specifically the High Dependency Unit, Coronary Care Unit, and Cardiac Intervention Unit. Staffed by approximately 80 registered nurses, these units provide critical care for patients with acute cardiac conditions, including those requiring complex interventions and invasive procedures.

### Data collection procedure

The pre-test was conducted to assess participants’ baseline knowledge and skills before the intervention. The first section gathered demographic information, including age, gender, years of experience, area of practice, and confidence level. The second section comprised 25 image-based questions on ECG strips, requiring participants to identify arrhythmias. Immediately following the pre-test, the educational intervention, a SDLpackage accessed through an online platform, was provided. To ensure that the SDL package and the pre- and post-tests accurately measured the intended learning outcomes, a panel of academic and clinical experts reviewed the materials and confirmed their construct validity, strengthening confidence in their internal reliability. The package comprised six lessons covering foundational ECG interpretation knowledge. This included understanding cardiac electrical activity, recognizing normal heart rhythms, identifying ECG waveform components, mastering lead placement, exploring common heart rhythm abnormalities, and understanding their underlying mechanisms and management strategies. A brief exercise concluded each lesson to assess participant knowledge. Two weeks after implementing the post-SDL package, a post-test was conducted in the Cardiology unit. Participants were required to complete the test within 45 minutes. The test included 25 image-based questions featuring ECG strips, designed to assess participants’ ability to identify various types of arrhythmias, ranging from basic to advanced difficulty levels.

### Outcome measures

The primary outcome measure was the improvement in ECG interpretation knowledge and skills as assessed by the pre- and post-test scores. Secondary outcomes included participants’ self-confidence in identifying arrhythmias under time constraints, simulating real clinical scenarios where nurses need to respond swiftly and accurately. Confidence levels were measured using a 4-point Likert scale, ranging from “very confident” to “unconfident. At each of pre and post stage the participants have to complete the questionnaire in a maximum of forty-five-minute minutes with one and a half minutes allowance for answering each item of ECG strips.

### Ethical considerations

Ethical approval for the study was obtained from the Dubai Scientific Research Ethics Committee (DSREC) under the Dubai Health Authority. All participants provided written informed consent and were informed of their right to withdraw from the study at any time. Continuing professional development (CPD) points were offered as compensation for their time and participation.

### Data analysis

Data were analyzed using SPSS version 28.0. Paired t-tests were conducted to compare the pre- and post-test scores, with a significance level set at p < 0.05. Descriptive statistics such as mean, standard deviation, frequency, and percentage were used to describe demographic variables and test scores. The Mann-Whitney test was used to analyze the relationship between gender and knowledge improvement, while One-Way ANOVA tested the association between years of experience and improvement in test scores. Additionally, the Kruskal-Wallis test was employed to assess differences in knowledge improvement based on the area of practice.

## Result

### Demographics of the participants

A total of 50 staff nurses from the Cardiology Units of Rashid Hospital participated in the study. The sample included nurses working in three primary areas: Coronary Care Unit (40%, n = 20), Cardiac Catheterization Lab (28%, n = 14), and General Cardiology Unit (32%, n = 16). The ages of participants ranged from 25 to 52 years, with a median age of 34.5 years. The majority of participants were female (70%, n = 35), and 52% (n = 26) had less than five years of experience in their respective units. Regarding educational attainment, almost all participants (98%) held a bachelor’s degree, with only 2% having completed diploma-level education. Most participants (46%, n = 23) rated themselves as only “somewhat confident” in ECG interpretation before the intervention. Detailed demographic data are provided in Table 1.

**Table 1:** Description of demographic variables of study subjects (n = 50).

Sample Characteristic	Frequency	Percentage %
Age		
*25-30*	8	16
*31-35*	17	34
*36-40*	14	28
*41-45*	5	10
*46-50*	5	10
*>50*	1	2
Gender		
*Male*	15	30
*Female*	35	70
Area of work		
*CIU*	14	28
*General Cardiology*	16	32
*CCU*	20	40
Years of experience		
*1-5 years*	26	52
*6-10 years*	15	30
*>11 years*	9	18
Educational Qualification		
*Bachelors*	49	98
*Diploma*	1	2
Confidence level		
*Very Confident*	3	6
*Somewhat confident*	23	46
*Neutral*	21	42
*Somewhat unconfident*	3	6

### Knowledge improvement

Following the self-directed learning (SDL) intervention, there was a clear and statistically significant improvement in nurses’ ECG interpretation knowledge. As shown in **Table 2**, paired t-test results revealed a **15.92% increase** in mean post-test scores compared to pre-test scores (from **47.12 to 63.04**, *p* < 0.001).

**Table 2:** Relation between knowledge in pre- and post-intervention (n = 50).

	Pre-Test	Post-Test	P-value
Total Knowledge	**47.12**	**63.04**	**<0.001**
Gender			
*Male*	44.26	57.06	0.013
*Female*	48.34	65.60	<0.001
Area			
*CCU*	49.0000	64.0000	0.002
*CIU*	47.7143	59.1429	0.018
*HDU*	44.2500	65.2500	<0.001
Year of Experience			
*1-5 yrs*	41.3846	63.2308	<0.001
*6-10 yrs*	51.2000	63.2000	0.010
*11 yrs and above*	56.8889	62.2222	0.257
Confidence level			
*Very Confident*	41.3333	50.6667	0.398
*Somewhat confident*	50.7826	67.8261	<0.001
*Neutral*	43.4286	59.4286	<0.001
*Somewhat unconfident*	50.6667	64.0000	0.063

### Gender and knowledge improvement

Although mean scores improved for both male and female participants after the intervention, female nurses had slightly higher post-test scores (65.60) compared to their male counterparts (57.06). However, when analyzed using the Mann-Whitney U test (due to the relatively small number of male participants), the difference in knowledge gain between genders was not statistically significant (*p* = 0.234).

### Years of experience and knowledge improvement

One of the more notable findings was the relationship between years of clinical experience and knowledge improvement. A one-way ANOVA revealed a statistically significant association (*p* = 0.020). Nurses with 1–5 years of experience showed the most marked improvement, with their mean score increasing from 41.38 to 63.23, a gain of nearly 22 percentage points. Nurses with 6–10 years of experience also demonstrated a significant improvement (*p* = 0.010). Interestingly, those with 11 years or more showed only a modest increase, which was not statistically significant (*p* = 0.257).

### Area of practice and knowledge improvement

When comparing knowledge gains by unit (CCU, CIU, and HDU), all groups showed statistically significant improvements in their mean scores. However, when analyzed collectively, no statistically significant difference was found between the groups in terms of knowledge improvement (*p* = 0.139) (Table 2).

### Confidence level and knowledge improvement

Confidence levels were also explored in relation to knowledge gains. Interestingly, nurses who reported being “somewhat confident” or “neutral” showed the most significant improvements after the intervention (p < 0.001). On the other hand, those who were “very confident” before the training showed only minimal improvement, and this was not statistically significant (p = 0.398). This finding may reflect a ceiling effect, where individuals who already perceive themselves as highly confident may have had less room for measurable improvement, or possibly overestimated their pre-intervention skills.

## Discussion

Accurate interpretation of electrocardiograms (ECGs) is an essential skill for nurses, particularly in acute and critical care settings, where prompt identification of arrhythmias and other cardiac abnormalities can directly impact patient outcomes. The results of this study confirm the critical importance of targeted educational interventions, such as the self-directed learning [SDL] package, in enhancing nurses’ proficiency in ECG interpretation. This finding aligns with previous research that highlights the significance of continuous education and training in improving clinical skills among healthcare professionals [2,7,12,13].

In this study, nurses demonstrated a significant improvement in ECG interpretation skills following the implementation of the SDL package, a result that is consistent with other studies that evaluated the effectiveness of educational interventions on ECG competency [9,12].. The improvement in post-test scores reflects the effectiveness of SDL as a flexible and accessible learning format that can be integrated into the busy schedules of nurses working in high-demand environments such as cardiology and critical care units.

The use of SDL in this study allowed nurses to learn at their own pace, reinforcing their ability to identify both common and complex arrhythmias. This aligns with findings from previous research showing that e-learning and self-directed educational methods are effective in enhancing the knowledge and practical skills of healthcare professionals across various disciplines [2,7,8,14] The flexibility of SDL enables learners to engage with the material in a manner that suits their individual needs, facilitating better retention of critical knowledge.

However, it is important to note that while the majority of nurses in this study demonstrated significant knowledge gains, the study also revealed a correlation between years of experience and improvement in ECG interpretation skills. Nurses with 1–5 years of experience showed the greatest improvement, whereas those with more than 11 years of experience exhibited the least improvement. This may suggest that nurses with more experience are more likely to rely on established patterns of practice and may benefit from more advanced or specialized training [4,15] Conversely, the absence of a significant correlation between gender and area of practice and knowledge improvement implies that the SDL package is broadly applicable across diverse nursing demographics and cardiology unit assignments [12,13]

The use of a wide range of ECG tracings with varying levels of difficulty was a methodological strength of this study, simulating real-life clinical conditions where nurses must rapidly interpret ECGs in high-pressure environments. This approach mirrors the complexity of clinical practice, providing participants with exposure to both common and rare arrhythmias, thereby enhancing their ability to respond to a variety of clinical scenarios. Such comprehensive training is crucial in preparing nurses for the challenges of critical care, where the timely and accurate identification of arrhythmias can be lifesaving [3,4]

Despite the positive outcomes, this study has limitations that must be acknowledged. The sample size of 50 nurses, although sufficient for this setting, limits the generalizability of the results. Future studies with larger and more diverse populations across multiple healthcare institutions would provide more robust evidence of the effectiveness of SDL in ECG education. Additionally, this study focused on short-term knowledge improvement, and it did not assess the long-term retention of ECG interpretation skills. Further research is needed to evaluate the durability of the educational intervention and its impact on clinical practice over time [6]. Moreover, while confidence was measured as a secondary outcome to gain insight into participants’ self-perceived competence, we acknowledge that self-reported confidence may be influenced by social desirability bias

### Implications for Nursing Practice

The findings of this study have several important implications for nursing practice. First, the significant improvement in ECG interpretation skills following the SDL intervention suggests that this learning format can be an effective tool for enhancing clinical competencies in nursing. Given the demanding nature of critical care environments, where nurses are often the first responders to life-threatening arrhythmias, the flexibility and accessibility of SDL offer a practical solution for continuous professional development without disrupting clinical duties [2,9].

Second, the results highlight the need for ongoing education and training in ECG interpretation, particularly for nurses with more years of experience. Tailoring educational interventions to address the specific needs of different experience levels could optimize learning outcomes and ensure that all nurses, regardless of their tenure, achieve the necessary proficiency in ECG interpretation [12,13]. Moreover, regular refresher courses and assessments could help sustain the skills acquired through SDL and e-learning programs, ensuring that nurses remain competent in their ECG interpretation skills throughout their careers.

Finally, this study underscores the potential for broader implementation of SDL and e-learning modules in nursing curricula. As nurses take on increasingly autonomous roles in patient care, particularly in high-stakes environments like emergency and critical care units, it is essential to equip them with the tools needed to make independent, informed clinical decisions. The success of the SDL package in this study suggests that similar educational approaches could be used to enhance nurses’ competencies in other critical diagnostic areas, ultimately improving patient care and outcomes [3,4].

## Conclusion

This study demonstrates the effectiveness of a self-directed learning (SDL) package in significantly improving nurses’ knowledge and skills in ECG interpretation, particularly in identifying arrhythmias. The improvement in post-test scores provides strong evidence that SDL is an effective educational tool, particularly for nurses working in critical care settings where quick and accurate diagnostic skills are essential. While the results are promising, further research is needed to explore the long-term retention of knowledge and the application of these skills in clinical practice. Additionally, expanding the study to include a larger and more diverse population would help validate the findings and support the wider adoption of SDL in nursing education.

In addition, this study highlights the potential of SDL as a flexible, accessible, and impactful tool for enhancing nurses’ clinical skills, particularly in cardiology and critical care settings. As healthcare continues to evolve, incorporating SDL into ongoing education programs will be essential for ensuring that nurses remain competent and confident in their ECG interpretation abilities, ultimately improving patient care and outcomes [2,9,12].
